# Single nucleotide polymorphisms in toll-like receptor genes and case-control association studies with bovine tuberculosis

**DOI:** 10.14202/vetworld.2016.458-464

**Published:** 2016-05-09

**Authors:** Ashish Bhaladhare, Deepak Sharma, Amit Kumar, Arvind Sonwane, Anuj Chauhan, Ranvir Singh, Pushpendra Kumar, Ramji Yadav, Mohd Baqir, Bharat Bhushan, Om Prakash

**Affiliations:** Division of Animal Genetics, Indian Veterinary Research Institute, Izatnagar, Uttar Pradesh, India

**Keywords:** bovine tuberculosis, immune response, resistance, single nucleotide polymorphisms, toll-like receptors

## Abstract

**Aim:**

Toll-like receptor 2 (TLR2) and TLR4 genes play critical roles in host recognition of *Mycobacterium bovis* infection and initiation of innate and adaptive immune response. The present study was aimed at exploring the association of seven single nucleotide polymorphisms (SNPs) in TLR2 and TLR4 genes with susceptibility/resistance against bovine tuberculosis (bTB) infection in cattle.

**Materials and Methods:**

A case-control resource population of 35 positive and 45 negative animals was developed after screening with single intradermal tuberculin test for bTB. Resource population was screened for SNPs in TLR2 and TLR4 genes using polymerase chain reaction-restriction fragment length polymorphism. The PROC LOGISTIC procedure of SAS 9.3 was used to find an association of allelic and genotypic frequencies with bTB.

**Results:**

In TLR2 gene, two of SNPs under study (rs55617172 and rs68268253) revealed polymorphism while in the case of TLR4 gene all four SNPs under investigation (rs8193041, rs207836014, rs8193060, and rs8193069) were found to be polymorphic in case-control population. SNP locus rs55617172 in TLR2 gene was found significantly (p<0.01) associated with susceptibility/resistance to TB in cattle.

**Conclusion:**

These findings indicate the presence of SNPs in TLR2 and TLR4 genes in our resource population. Upon validation in independent, large resource population and following biological characterization, SNP rs55617172 can be incorporated in marker panel for selection of animals with greater resistance to bTB.

## Introduction

Bovine tuberculosis (bTB) is a chronic debilitating infectious disease of cattle caused by *Mycobacterium bovis*. In addition to being an important pathogen of cattle, the host range of *M. bovis* has been reported to include humans, non-human primates, goats, pigs, buffalo, badgers, possums, deer, and bison [1,2]. bTB has been reported in almost every country of the world but is endemic in Africa and Indian subcontinent [3]. It costs estimated $3 billion annually in global agricultural losses [4] and is the fourth most important livestock disease worldwide [3]. A loss of 10-25% in milk production efficiency has been reported in TB-infected animals [5,6].

*M. bovis* infection has a chronic progression where pathogenesis begins with bacterial entry to host lungs by inhalation and bacteria phagocytosis by alveolar macrophages. When disease develops over a period with incubation period ranging from several weeks to months, the associated granulomatous pathological changes are seen mainly in the lower and upper respiratory tract along with apparent clinical symptoms [7]. Thus, cattle may become infectious long before they exhibit clinical signs or lesions. Consequently, the mainstay of TB control in cattle lies in the early detection and removal of infected animals. Currently, no effective vaccine exists for bTB and treatment regimen available is not cost effective and generally not recommended. Moreover, eradication of the disease by slaughtering of affected animals is also difficult because of the socio-economic condition of farmers and the social customs or religious taboos [8]. Given the difficulties in eradicating bTB by conventional measures, improving genetic resistance to diseases among livestock populations could be an effective alternate strategy as the genetic gain is cumulative and permanent. Genetic variation in susceptibility to bTB has been observed in cattle. Differences in susceptibility to bTB at the level of the genus have been reported by Ameni *et al*. [9], indicating that *Bos indicus* cattle are more resistant than *Bos taurus*. Significant heritability has been reported for susceptibility to bTB in Holstein cattle in the UK [10] and in the Republic of Ireland [11].

Candidate gene approach can serve as a useful tool in identifying resistant superior genotypes for the production of the new resistant animal population [12]. Toll-like receptor 2 (TLR2) and TLR4 genes codes for molecules that are localized on the cell surface and recognize mycobacterial membrane components [13]. They recruit a specific set of adaptor molecules such as MyD88 and TRIF and activate MyD88-dependent and TRIF-dependent signaling pathways that lead to the secretion of inflammatory cytokines, type I interferon (IFN), chemokines, and antimicrobial peptides [14]. These responses cause recruitment of neutrophils, activation of macrophages, and induction of IFN-stimulated genes, resulting in the direct killing of the infected mycobacterial pathogens and the induction of adaptive immunity [15]. TLR2 and TLR4 genes are thus promising candidates for exploring the association with susceptibility/resistance to bTB. Association of TLR2 polymorphism with TB has been reported in human [16-18], and in the case of Cattle, SNPs in TLR2 and TLR4 genes have been associated with susceptibility to paratuberculosis infection [19,20]. Polymorphisms have been reported within the TLR 2 and TLR4 genes in cattle [21,22]; however, reports of marker level association studies with bTB in cattle are lacking, especially indigenous breeds that could be used for selection and breeding of resistant/tolerant animals. Therefore, the objectives of this study were to genotype a resource population tested for bTB infection and to evaluate the association of seven single nucleotide polymorphisms (SNPs) in the TLR2 and TLR4 genes for susceptibility/resistance to bTB in cattle.

## Materials and Methods

### Ethical approval

The experiment was prior approved by the Animal Ethics Committee of the Institute constituted as per the article number 13 of the CPCSEA rules laid down by Government of India and conducted following the code of ethics for animal experimentation.

### Development of case-control population

For the current investigation, resource population comprised 245 cattle including Indigenous (Koshi, Sahiwal, Gir)/Non-descript and crossbred from Shri Mataji Gaushala, Barsana. All animals were maintained under similar feeding and managemental practices and had an equal opportunity of infection. Animals were screened for the presence of bTB by Single intradermal tuberculin test, wherein increase in thickness of skin after 72 h of intradermal injection of tuberculin antigen was noted to develop case (tuberculin positive) and Control (tuberculin negative) resource panel. An intradermal inoculation of 0.1 ml of tuberculin PPD antigen on neck region was carried out. The skin thickness was measured with vernier calipers before and 72 h after inoculation. Based on thickness, cattle were classified into three groups: Those showed marked swelling and skin thickness more than 4 mm (positive), skin thickness <4 mm and >2 mm (inconclusive), and no reaction >2 mm (negative). The inconclusive animals were not included in the present investigation. A case and control resource panel of 35 positive and 45 negative animals was developed.

### Sample collection and isolation of genomic DNA

From each of case and control animals, 5 ml of blood was collected from a jugular vein in tubes containing 2.7% EDTA and stored at −20°C. DNA was isolated from whole blood using Promega Wizard^®^ Genomic DNA Purification Kit as per recommended protocols. The DNA concentration was determined using Qubit Fluorometer. DNA quality was also assessed by 1% submarine agarose gel electrophoresis. 1µl of genomic DNA was resolved on 1% agarose gel stained with ethidium bromide or SYBR^®^ Safe DNA gel stain, and quantification was made by comparing the intensity of the band with the intensity of a known quantity of lambda DNA. Only thick DNA band and without smearing were chosen for further processing.

### Genotyping of SNPs

Primers for the 7 SNPs in TLR2 (rs55617172, rs43706434, and rs68268253) and TLR4 (rs8193041, rs207836014, rs8193060, and rs8193069) genes were designed using OligoAnalyzer (Integrated DNA Technology software) software for amplification of the loci. The detail of primers and restriction enzymes are being presented in Table-1. Concerned amplicons were amplified under the optimized polymerase chain reaction (PCR) condition. The PCR reaction was carried out in 25 µl volume which included 1 µL of each primer (forward and reverse), 1.5 µL MgCl_2_, 5 µl buffer, 0.2 µL dNTPs, 0.125-0.25 µl Taq polymerase, 1 µl genomic DNA, and Nuclease-free water 15.05-15.175 µl. The cycling program used for amplification having following steps: Initial denaturation (94°C for 4 min), followed by 35 cycles of 30 s at 94°C, 30 s at annealing temperature (Table-1), 30 s at 72°C, and final extension of 5 min at 72°C. The PCR products were resolved in 2.4% agarose gel and visualized under UV light after staining with ethidium bromide. Restriction digestion was carried out in 25 µL reaction volume which included 20 µL of PCR product, 1.5 U of restriction enzyme, 2.5 µL of 10× buffer and NFW to make the volume up to 25 µL and incubated at recommended temperature as prescribed by the manufacturer for 16 h. The restriction enzyme digestion was made at the optimized conditions and the restriction digested products were resolved in 3.5% agarose gel and visualized under UV light after staining with ethidium bromide. Mass genotyping of all case-control resource population for all 7 SNPs was done using PCR-restriction fragment length polymorphism (PCR-RFLP).

**Table-1:** SNPs with their primers and restriction enzymes.

SNP id	Gene	SNP	Position	Primer sequence (5’-3’)	Annealing temperature (°C)	Restriction enzyme	Amplicon size (bp)	Fragment size (bp)
rs55617172	TLR2	G/T	+385	TTAAACTCCATCCCCTCTGG TAAAGGGACCTGAACCAGG	59.2	EcoRV	245	63, 182
rs43706434	TLR2	G/A	+651	GAACCTGAAGACTCTGAGG CTGATCTCAAGCTCCTCAAG	54.0	PstI	318	224, 94
rs68268253	TLR2	G/T	+1141	ACCAAGGTTTGAAGGAAGGG TGATGACTGTACCCATGATGG	56.8	BsaAI	327	235, 92
rs8193041	TLR4	C/T	+480	CGTAACCCAGCACTGCTTTG GCCTGTTAATGCCCTGTAACC	58.5	BstUI	405	159, 246
rs207836014	TLR4	C/T	+869	CACCTCTCCACCTTGATACTG CTTCGCAGAGTCAATCCATTG	56.5	HaeIII	398	92, 306
rs8193060	TLR4	C/T	+2126	GCTTAGTCAGTCTGCAAACC CACTGCAGGAAACTCTGATG	55.8	ApeKI	373	247, 126
rs8193069	TLR4	C/T	±2491	GGGTCCTAGTCTACAAGTTC ACGATGAAGATGATGCCAG	53.9	BsiHKAI	367	75, 292

All above 7 SNPs were taken from SNP Database-dbSNP (http://www.ncbi.nlm.nih.gov/projects/SNP/). SNP=Single nucleotide polymorphisms, TLR=Toll-like receptor

### Statistical analysis

Initially in univariate logistic regression analysis, the non-genetic factors such as age (two levels), sex (two levels), and breed (two levels) were fitted and found that none of these effects were significantly affecting the single intradermal tuberculin test result. The association between various allelic variants with bTB tolerance/susceptibility was worked out by suitable statistical techniques using different procedures of SAS 9.3. The PROC LOGISTIC procedure of SAS 9.3 was used to find the association of allelic and genotypic frequencies with bTB. The odds ratio (OR) of genotypes was calculated in affected population versus their contemporary genotypes. The PROC ALLELE procedure of the SAS 9.3 used for the estimation of polymorphism information content (PIC), Hardy-Weinberg equilibrium (HWE), and heterozygosity.

## Results and Discussion

**Figure-1:**
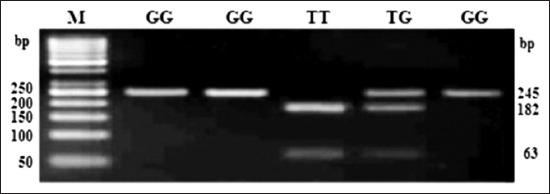
Polymerase chain reaction-restriction fragment length polymorphism profile of single nucleotide polymorphism rs55617172 in 3.5% agarose gel. Lane M: 50 bp ladder.

**Figure-2:**
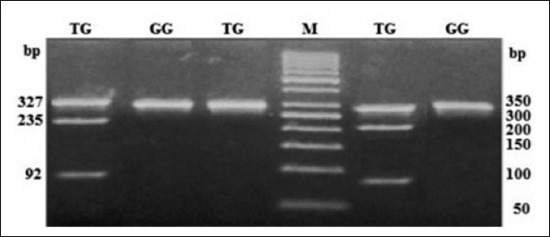
Polymerase chain reaction-restriction fragment length polymorphism profile of single nucleotide polymorphism rs68268253 in 3.5% agarose gel. Lane M: 50 bp ladder.

**Figure-3:**
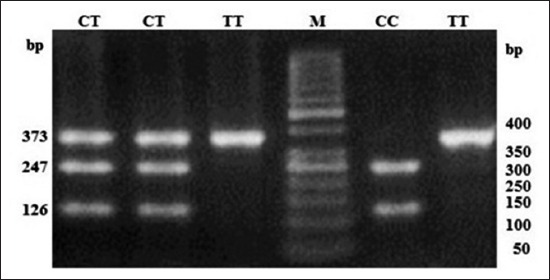
Polymerase chain reaction-restriction fragment length polymorphism profile of single nucleotide polymorphism rs8193060 in 3.5% agarose gel. Lane M: 50 bp ladder.

**Figure-4:**
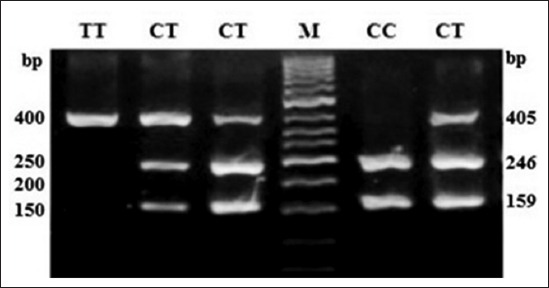
Polymerase chain reaction-restriction fragment length polymorphism profile of single nucleotide polymorphism rs8193041 in 3.5% agarose gel. Lane M: 50 bp ladder.

**Figure-5:**
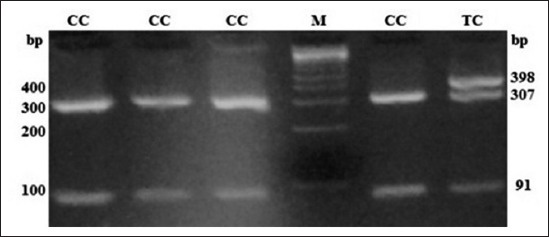
Polymerase chain reaction-restriction fragment length polymorphism profile of single nucleotide polymorphism rs207836014 in 3.5% agarose gel. Lane M: 100 bp ladder.

**Figure-6:**
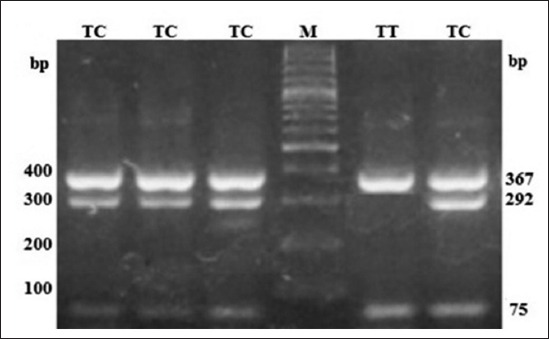
Polymerase chain reaction-restriction fragment length polymorphism profile of single nucleotide polymorphism rs8193069 in 3.5% agarose gel. Lane M: 100 bp ladder.

All non-genetic factors (breed, age, and sex) had non-significant (p<0.05) effect on the single intradermal tuberculin test for bTB. In the case of TLR2 gene, two of SNPs under study (rs55617172 and rs68268253) revealed polymorphism (Figures-1 and 2), whereas monomorphism was found in SNP rs43706434. While in the case of TLR4 gene all four SNPs under investigation (rs8193041, rs207836014, rs8193060, and rs8193069) were found to be polymorphic (Figures-3-6). These findings indicate the presence of these SNPs in our resource population. PIC ranged from 0.1186 (rs68268253) to 0.3744 (rs8193069) and revealing low to moderate polymorphism at the tested SNPs. The heterozygosity varied widely from a very low estimate of 0.024 (rs207836014) to 0.952 (rs8193069). Similarly, allelic diversity ranged from 0.023 (rs207836014) to 0.4989 (rs8193069). Except for SNP rs8193060 and rs207836014, all other SNPs in the population departed from HWE significantly (p<0.05). This significant departure from HWE may be due to non-static herd strength at the Gaushala. The allelic frequencies and the genotypic frequencies in case and control populations at different SNP loci and their effect on susceptibility to infection along with OR have been shown in Tables-2 and 3, respectively. For the polymorphic SNPs, PIC, heterozygosity, Allelic diversity and probabilities of being the population in HWE is presented in Table-4.

**Table-2:** Allelic frequency distribution of TLR genes and their association with bTB.

SNP	Allele	Allele frequency		p value	OR (95% CI)

		Case	Control		
rs55617172	G	60 (0.857)	70 (0.714)	<0.01	3.60 (1.47-8.81)
	T	10 (0.143)	28 (0.286)		1
rs68268253	G	66 (0.943)	92 (0.939)	0.91	1.08 (0.29-3.96)
	T	4 (0.057)	6 (0.0612)		1
rs8193041	C	44 (0.629)	58 (0.592)	0.63	1.16 (0.62-2.19)
	T	26 (0.371)	40 (0.401)		1
rs207836014	C	69 (0.986)	97 (0.990)	0.76	0.71 (0.04-11.56)
	T	1 (0.014)	1 (0.010)		1
rs8193060	C	12 (0.171)	23 (0.235)	0.32	0.67 (0.31-1.46)
	T	58 (0.829)	75 (0.765)		1
rs8193069	C	33 (0.471)	47 (0.480)	0.92	0.96 (0.52-1.78)
	T	37 (0.529)	51 (0.520)		1

SNP=Single nucleotide polymorphisms, TLR=Toll-like receptor, bTB=Bovine tuberculosis, OR=Odds ratio, CI=Confidence interval

**Table-3:** Genotype frequency distribution of TLR genes and their association with bTB.

SNP	Genotype	Genotype frequency		p value	OR (95% CI)

		Case	Control		
rs55617172	GG	27 (0.772)	26 (0.531)	<0.01	2.88 (0.52-16.14)
	TG	6 (0.171)	18 (0.367)		0.42 (0.05-3.22)
	TT	2 (0.057)	5 (0.102)		1
rs68268253	G/G	31 (0.886)	43 (0.878)	0.91	1.08 (0.28-4.16)
	G/T	4 (0.114)	(0.122)		1
rs8193041	C/C	10 (0.286)	9 (0.184)	0.21	<0.01 (<0.01->999.99)
	C/T	24 (0.686)	40 (0.816)		<0.01 (<0.01->999.99)
	T/T	1 (0.029)	0		1
rs207836014	C/C	34 (0.971)	48 (0.980)	0.81	0.71 (0.04-11.72)
	C/T	1 (0.029)	1 (0.020)		1
rs8193060	C/C	2 (0.057)	3 (0.061)	0.48	0.77 (0.12-5.01)
	C/T	8 (0.229)	17 (0.347)		0.55 (0.2-1.48)
	T/T	25 (0.714)	29 (0.592)		1
rs8193069	C/T	33 (0.943)	47 (0.959)	0.73	0.7 (0.09-5.24)
	T/T	2 (0.052)	2 (0.041)		1

TLR=Toll-like receptor, bTB=Bovine tuberculosis, OR=Odds ratio, CI=Confidence interval

**Table-4:** PIC, heterozygosity and HWE and probability distribution in total resource population of cattle.

SNP	PIC	Heterozygosity	Allelic diversity	HWE (χ²)
rs55617172	0.2755	0.2500	0.3299	0.0265
rs68268253	0.1186	0.1190	0.1237	<0.0001
rs8193041	0.3633	0.7619	0.4770	<0.0001
rs207836014	0.0232	0.0238	0.0235	0.9121
rs8193060	0.2755	0.2976	0.329	0.3703
rs8193069	0.3744	0.9524	0.4989	<0.0001

PIC=Polymorphism information content, HWE=Hardy-Weinberg equilibrium

At SNP locus rs55617172, two alleles, i.e. G and T and three genotypes, i.e. GG (245 bp), TT (63 and 182 bp), and TG (245, 63, and 182 bp) were observed. The probability values showed that the genotype (p<0.01) as well as allele (p<0.01) had a significant effect on the occurrence of bTB. The OR of G versus T was 3.60 (1.47-8.81; 95% confidence interval [CI]), whereas OR of GG versus TT and TG versus TT was 2.88 (0.52-16.14; 95% CI) and 0.42 (0.05-3.22; 95% CI), respectively (Tables-2 and 3). At SNP locus rs68268253, two alleles, i.e. G and T and two genotypes, i.e. GG (327 bp) and TG (327, 235, and 92 bp) were observed. The probability values showed that the genotype (p=0.91) as well as allele (p=0.91) had a non-significant effect on the occurrence of bTB. The OR of G versus T was 1.08 (0.29-3.96; 95% CI), whereas OR of GG versus TG was 1.08 (0.28-4.16; 95% CI), respectively (Tables-2 and 3). At SNP locus rs8193060, two alleles, i.e. C and T and three genotypes, i.e. CC (247, 126 bp), TC (373, 247, and 126 bp), and TT (373 bp) were observed. The probability values showed that the genotype (p=0.48) as well as allele (p=0.32) did not have any significant effect on the occurrence of bTB. The OR of C versus T was 0.67 (0.31-1.46; 95% CI), whereas the OR of CC versus TT and TC versus TT was 0.77 (0.12-5.01; 95% CI) and 0.55 (0.2-1.48; 95% CI), respectively (Tables-2 and 3). At SNP locus rs8193041, two alleles, i.e. C and T and three genotypes, i.e. CC (159 and 246 bp), TC (405, 159, and 246 bp), and TT (405 bp) were observed. The probability values showed that the genotype (p=0.21) as well as allele (p=0.63) had not significant effect on the occurrence of bTB. The OR of C versus T was 1.16 (0.62-2.19; 95% CI), whereas OR of CC versus TT and TC versus TT was <0.01 (<0.01->999.99; 95% CI) and <0.01 (<0.01->999.99; 95% CI), respectively (Tables-2 and 3). At SNP locus rs207836014, two alleles, i.e. C and T and two genotypes, i.e. CC (91 and 307 bp) and TC (398, 91, and 307 bp) were observed. The probability values showed that the genotype (p=0.81) as well as allele (p=0.76) had not significant effect on the occurrence of bTB. The OR of C versus T was 0.71 (0.04-11.56; 95% CI), whereas OR of CC versus TC was 0.71 (0.04-11.72; 95% CI), respectively (Tables-2 and 3). At SNP locus rs8193069, two alleles, i.e. C and T and two genotypes, i.e. TC (367, 75 and 292 bp) and TT (367 bp) were observed. The probability values showed that the genotype (p=0.73) as well as allele (p=0.92) had not significant effect on the occurrence of bTB. The OR of C versus T was 0.96 (0.52-1.78; 95% CI), whereas OR of TC versus TT was 0.7 (0.09-5.24; 95% CI), respectively (Tables-2 and 3). The linkage disequilibrium analysis revealed that the significantly linked loci/SNPs were TLR4-C2126T with TLR2-G1141T. All other loci/SNPs of the investigation were having non-significant LD suggested that there was no linkage between alleles of these loci.

Overall, out of seven SNPs explored in TLR2 and TLR4 genes for association with susceptibility/resistance to bTB in our case: Control resource population rs55617172 in TLR2 gene showed a significant effect on susceptibility to bTB (p<0.01). The OR of GG versus TT (Table-3) revealed more susceptibility of GG genotype to bTB while the OR of TG genotype versus TT genotype suggested that TG genotypes are more resistant to bTB as compared to TT genotype. With regard to the effect of alleles, OR of G versus T allele (Table-2) suggested more susceptibility to bTB with G allele in comparison to T allele. In earlier studies, polymorphisms have been previously reported in TLR2 and TLR4 genes in cattle [21-24]. There is no report of the association of SNPs in TLR2 with the susceptibility to bTB. However, Zhang *et al*. [25] reported the association of TLR2-G385T with mastitis in cattle, wherein mean of genotype GG was reported significantly lower than those of genotype TT and TG for somatic cell score. However, there are also several reports on the association of TLR2 polymorphism with bTB in human [16-18]. Though all the four SNPs studied for TLR4 gene showed their presence in our case, control population but the polymorphism at these loci was not found to be significantly (p>0.05) associated with the susceptibility to bTB. Though TLR-4 have a specific role in immune response against bTB [26], the association of SNPs from TLR4 gene with susceptibility to bTB had not been reported in cattle. Since TLR4 have a key role in immunity against *M. bovis* thus more number of SNPs validations in a larger population may reveal some significant association of TLR4 SNPs with susceptibility of bTB.

## Conclusion

Evidence shows that genetic factors might be important determinants in explaining individual variation in the outcome of exposure to *M. bovis* pathogen in cattle sharing similar environmental and managemental conditions. Candidate genes coding for proteins with very specific and unique roles in immune responses are potential strong candidates for investigating the genetic basis of bTB susceptibility/resistance. TLR2 and TLR4 genes codes for important molecules involved in modulation of both innate and adaptive immune response against bTB. In the present investigation, we raised the question whether polymorphisms of TLR2 or TLR4 are associated with the risk of bTB. Out of the 6 existent SNPs in TLR2 and TLR4 genes in the case-control population, rs55617172 locus was found significantly (p<0.01) associated with susceptibility/resistance to TB in cattle. All the SNPs investigated were non-synonymous and suggested their functional role in the immune response against bTB. Additional validation in independent, large resource population and biological characterization are necessary before SNP can be incorporated in SNP panel for selection of animals with greater resistance to bTB. In addition, other SNPs of these concerned genes could be further exploited for association studies in cattle.

## Authors’ Contributions

AB: Research was done by this author as the part of his master’s degree thesis dissertation; DS: Designed the study and supervised the research as major advisor; RY, MB, and OP: Worked and collaborated in the lab work and compilation of the results as well as the manuscript; AK, AS, and AC: Provided valuable suggestions regarding the design of the experiment and analysis of the data collected during research; PK, RS, and BB: Advised in all aspects of the work and shared lab facilities. All authors read and approved the final manuscript.
